# Association between autoimmune diseases and atrial fibrillation: a real-world analysis from German outpatient data

**DOI:** 10.1186/s12872-026-05559-5

**Published:** 2026-02-02

**Authors:** Jamschid Sedighi, Mark Luedde, Priyanka Boettger, Boris Dinov, Birgit Assmus, Samuel Sossalla, Karel Kostev

**Affiliations:** 1 Medical Clinic I, Cardiology and Angiology, Justus-Liebig-University, Klinikstraße 33, Giessen, 35392 Germany; 2 Christian-Albrechts-University of Kiel, Kiel, Germany; 3 Department of Cardiology, Kerckhoff-Clinic, Bad Nauheim, Germany; 4 German Center for Cardiovascular Research (DZHK), Partner Site Rhine-Main, Frankfurt am Main, Germany; 5 Epidemiology, IQVIA, Frankfurt, Germany

**Keywords:** Atrial fibrillation, Autoimmune disease, Sex disparities, Real-world data

## Abstract

**Background:**

Atrial fibrillation (AF) is the most common sustained arrhythmia in clinical practice and is associated with considerable morbidity and mortality. Chronic systemic inflammation is increasingly recognized as a contributing factor in AF pathogenesis. Autoimmune diseases (AIDs), with their prototypical inflammatory conditions, may therefore be associated with AF. However, evidence from routine outpatient care regarding these potential associations across multiple AIDs remains limited.

**Objective:**

We aimed to examine whether autoimmune diseases are associated with increased odds of atrial fibrillation and to assess whether these associations differ between women and men.

**Methods:**

We conducted a retrospective 1:1 matched case-control study using data from the Disease Analyzer database (IQVIA). Adult patients with AF (2010–2024) were matched to AF-free controls based on age, sex, comorbidities, medication use and observation time. The presence of predefined AIDs prior to AF diagnosis was identified using ICD-10 codes. Conditional logistic regression was used to estimate odds ratios (ORs) and 95% confidence intervals (CIs) and sex-stratified data were analyzed.

**Results:**

A total of 174,992 AF cases and 174,992 matched controls were included (mean age 74.0 years, 48% female). Overall, any AID was associated with AF (OR 1.12; 95% CI 1.09–1.15). Significant associations were observed for rheumatoid arthritis (OR 1.14; 95% CI 1.10–1.18), inflammatory bowel disease (OR 1.16; 95% CI 1.06–1.27), psoriasis (OR 1.10; 95% CI 1.05–1.15), and Graves’ disease (OR 1.29; 95% CI 1.18–1.41).

**Conclusion:**

Several AIDs were modestly associated with atrial fibrillation in routine outpatient care. Sex-stratified analyses suggested variation in point estimates for some associations, while interaction analyses showed limited evidence for true sex-specific effect modification. These findings support the role of systemic inflammation in AF pathogenesis and underscore the need for interdisciplinary cardiovascular risk assessment in patients with autoimmune conditions.

## Introduction

Atrial fibrillation (AF) is the most common sustained cardiac arrhythmia and is associated with an increased risk of stroke, heart failure, and mortality worldwide [1, 2]. While conventional cardiovascular risk factors explain a substantial portion of AF incidence, chronic systemic inflammation has been increasingly recognized as a key contributor to atrial remodeling and arrhythmogenesis [3–5].

Autoimmune diseases (AIDs) encompass diverse conditions with distinct inflammatory profiles. Although many involve chronic immune activation, their inflammatory burden and therapeutic exposures (e.g., glucocorticoids or biologic agents) vary considerably and may influence cardiovascular risk, including AF [6]. Several recent large-scale studies have explored the link between AIDs and AF. A nationwide Danish registry study reported significantly increased long-term AF incidence across 28 autoimmune conditions, with an overall adjusted hazard ratio of 1.63 compared to the general population [7]. Similarly, a UK Biobank analysis found that AIDs were independently associated with higher AF risk, particularly among women [8].

These studies provide important evidence that autoimmune conditions are associated with AF, but they are based on registry or volunteer cohort data and do not fully reflect comorbidity profiles, prescribing patterns, and diagnostic practices in routine outpatient care. Moreover, they only partly addressed potential sex differences, although women and men differ in immune regulation, AID prevalence and AF susceptibility.

To address these gaps, we used a large, representative database of German general practices to examine the association between selected autoimmune diseases and newly diagnosed AF in routine outpatient care. Our primary aim was to quantify these associations after careful matching on major cardiovascular comorbidities and medications. A second, pre-specified aim was to explore whether the strength of these associations differs between women and men by conducting sex-stratified analyses.

## Methods

### Database

This study used data from the Disease Analyzer database (IQVIA). Details of this database have been published previously [9]. In brief, the Disease Analyzer database contains data on demographic variables, diagnoses and prescriptions of outpatients obtained in office-based practices in Germany. The database covers approximately 1300 general practices in Germany. It has previously been shown that the panel of practices included in the Disease Analyzer database is representative of office-based practices in Germany [9].

### Study population

The study population included adult patients (≥ 18 years) with a newly documented diagnosis of AF (I48.0, I48.1, I48.2, I48.9) between January 2010 and December 2024 (index date). To minimize misclassification of prevalent AF as incident, we required a continuous look-back period of at least 12 months before the index date with no prior AF diagnosis. Any patient with an AF code during this pre-observation period was excluded. Because AF subtype coding (paroxysmal, persistent, permanent) is not consistently available in outpatient records, all AF diagnoses were analyzed collectively. Controls were individuals without atrial fibrillation and flutter diagnoses in their history who were matched (1:1) by nearest neighbor propensity scores based on age, sex, pre-observation time prior to the index date in years, and diagnoses documented within 12 months prior to the index date including obesity (ICD-10: E66), type 2 diabetes mellitus (ICD-10: E11), dyslipidemia (ICD-10: E78), hypertension (ICD-10: I10), ischemic heart diseases (ICD-10: I20-I25), heart valve disorders (ICD-10: I08, I34-I39), heart failure (ICD-10: I50), chronic obstructive pulmonary disease (COPD) (ICD-10: J44), and nicotine dependence (ICD-10: F170). Furthermore, drug classes including beta-blockers, diuretics, calcium channel blockers, ACE inhibitors, angiotensin II receptor blockers, lipid-lowering drugs, systemic corticosteroids, thyroid hormones, and betaagonists prescribed within 12 months prior to the index date were included in the matching.

**Fig. 1:**
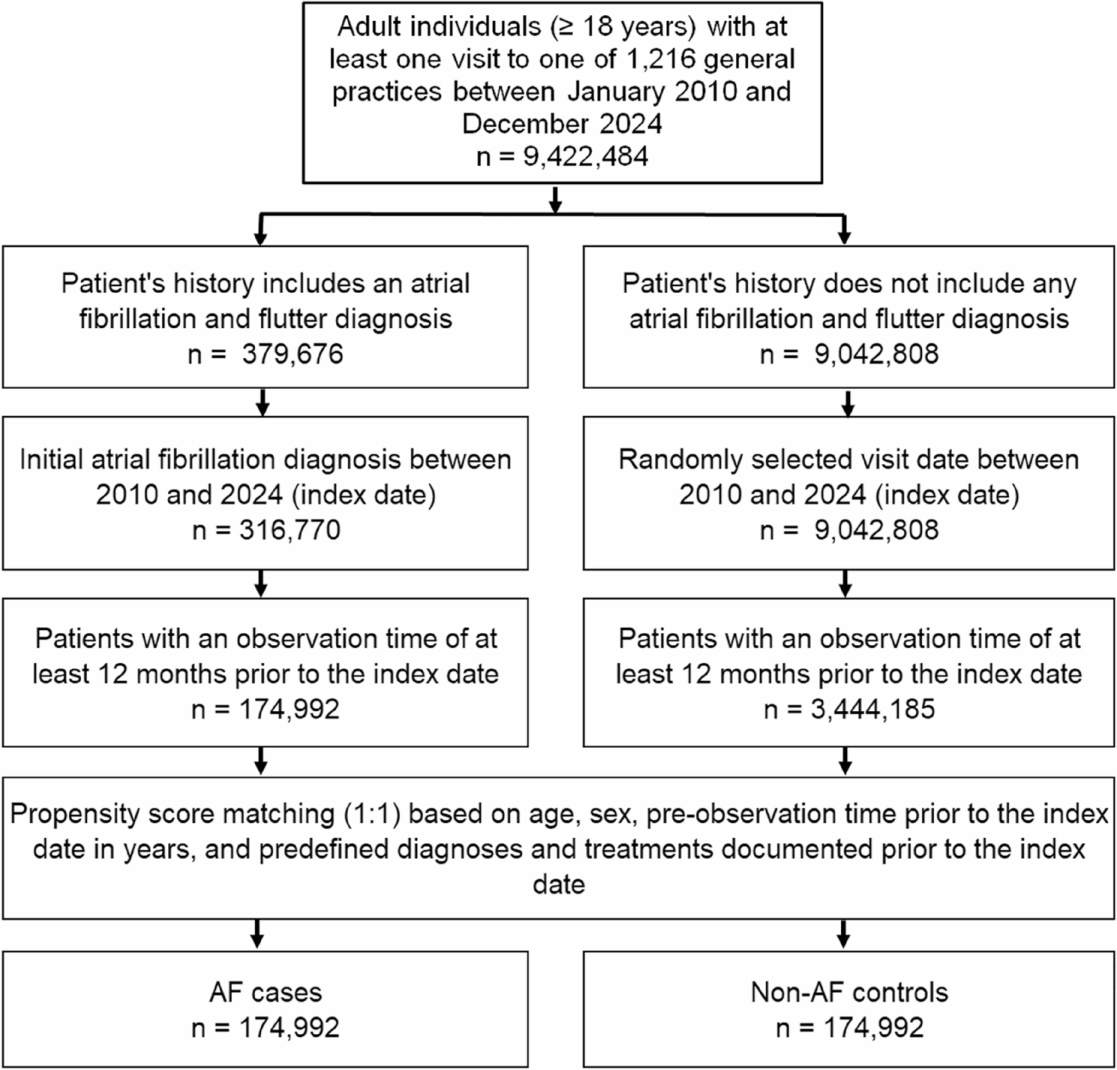
Selection of study patients

**Fig. 2:**
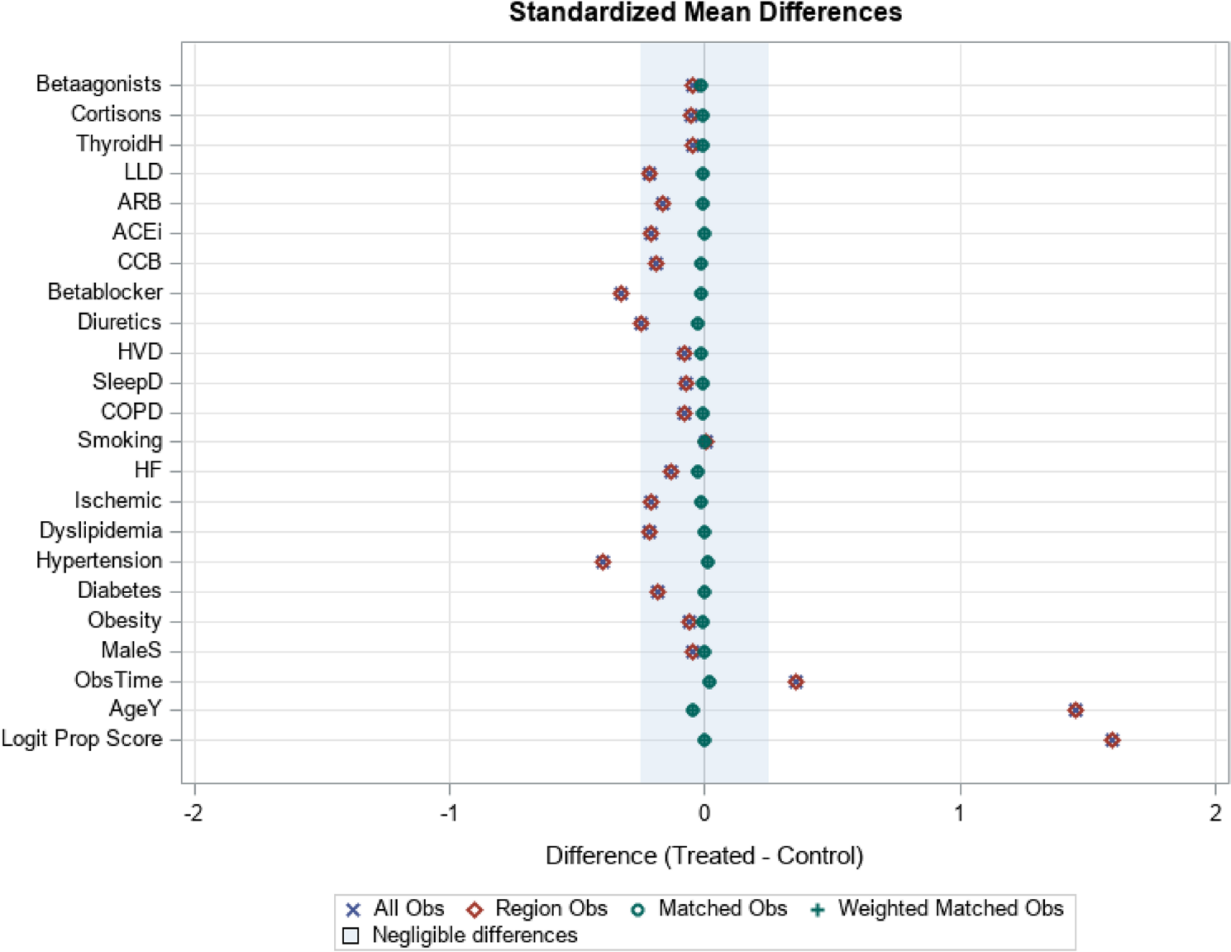
Assessment of covariate balance using standardized mean differences. This Love plot displays the standardized mean differences comparing AF cases and matched controls before matching (“All Obs”, blue crosses) and after 1:1 propensity score matching (“Matched Obs”, green circles). The shaded region represents the threshold for negligible imbalance (|SMD| < 0.1). Lower absolute SMD values indicate better covariate balance. Abbreviations: Betaagonists = β-agonists (bronchodilators), Cortisons = systemic corticosteroids, ThyroidH = thyroid hormone replacement therapy, LLD = lipid-lowering drugs, ARB = angiotensin II receptor blockers, ACEi = angiotensin-converting enzyme inhibitors, CCB = calcium channel blockers, HVD = heart valve disease, SleepD = sleep disorders, COPD = chronic obstructive pulmonary disease, HF = heart failure, Ischemic = ischemic heart disease, ObsTime = observation time prior to index date, AgeY = age in year, Logit Prop Score = logit of the propensity score. After matching, all covariates showed SMDs within the negligible imbalance range, indicating excellent balance between AF cases and controls

As tobacco use information is not available, we used diagnoses of nicotine dependence and COPD which might represent smoking behavior. For individuals without AF (controls), the index date was a randomly selected visit date between January 2010 and December 2024. The flow diagram of study participants is shown in Fig. 1. For propensity score matching, a standardized mean difference (SMD) of less than 0.1 was allowed, indicating that adequate covariate balance has been achieved (Fig. 2).

AIDs were identified using ICD-10 codes recorded by the treating general practitioner at any time prior to the index date. AIDs were defined as present if at least one corresponding ICD-10 code was documented in the patient’s history, and they were treated as ‘ever diagnosed’ before the AF index date. The date of AID onset was operationalized as the date of the first recorded ICD-10 code in the database and does not necessarily reflect the true clinical onset of disease. We did not distinguish incident from prevalent AID and information on disease duration, activity, or severity was not available.

### Study outcome

Outcome of the study was the association between autoimmune disorders documented in the complete patient history prior to the index date and subsequent AF diagnosis. The diagnoses of interest were type 1 diabetes (T1D) (ICD-10: E10) inflammatory bowel diseases (IBD) (ICD-10: K50, K51), rheumatoid arthritis (ICD-10: M05, M06), psoriasis (ICD-10: L40), systemic lupus erythematosus (SLE) (ICD-10: M32), autoimmune thyroiditis (ICD-10: E06.3), Graves’ disease (ICD-10: E05.0), multiple sclerosis (ICD-10: G35), ankylosing spondylitis (ICD-10: M45), and celiac disease (ICD-10: K90.0). AIDs were selected based on predefined criteria, including sufficient diagnostic frequency in the database to allow matched analyses, relevance to primary-care settings, and previously reported associations with cardiovascular or arrhythmic outcomes. Rare autoimmune conditions or those primarily diagnosed and managed in specialist settings were excluded because of very low case numbers and incomplete documentation. As a result, both systemic and organ-specific autoimmune diseases with adequate representation in the database were included.

### Statistical analyses

To examine whether each AID was associated with the presence of AF diagnoses, univariable conditional logistic regression models and estimated odds ratios (ORs) with 95% confidence intervals (95%CI) were utilized. This model was additionally calculated separately for female and male patients. To account for multiple testing, we applied a Bonferroni correction based on the full set of eight predefined regression models that formed the family of primary hypotheses in this study. These models included the associations between atrial fibrillation and any autoimmune disease in the overall population and in women and men separately, the associations between atrial fibrillation and each individual autoimmune disease in the overall population and in sex‑stratified analyses, and the interaction models evaluating sex in combination with each autoimmune disease as well as with the presence of any autoimmune disease. Because all eight models were considered part of the same hypothesis family, the Bonferroni‑adjusted significance threshold was defined as *p* < 0.0625, obtained by dividing the conventional α level of 0.05 by the number of primary models. The p‑values themselves were not modified; statistical significance was assessed relative to this corrected threshold.

All analyses were conducted using SAS version 9.4 (SAS Institute, Cary, NC, USA).

Given that patients were selected based on AF status and matched to controls, this study represents a retrospective matched case–control design. As such, the analysis estimates associations rather than prospective risk.

Our research protocol was evaluated by the local ethics committee of Christian-Albrechts-University Kiel (CAU), Germany. Since we used only anonymized data, which could not be traced back to individual persons, it was not necessary to obtain informed consent from individual patients (file reference D413/21 of the CAU ethics committee). All methods were performed in accordance with the relevant guidelines and regulations.

## Results

### Baseline characteristics

A total of 174,992 patients with newly diagnosed AF and 174,992 matched controls without AF were included in the analysis (Fig. 1). The mean age of the study population was 74.0 years (SD ± 12.1), and 48.1% were female. Baseline characteristics, including cardiovascular comorbidities and relevant medication use, were well balanced between AF cases and controls (Table 1).

**Table 1:** Characteristics of study patients after 1:1 matching

Variable	Patients with AF (*n* = 174,992)	Patients without AF (*n* = 174,992)	SMD
Age in years			
Mean (SD)	73.6 (11.9)	74.1 (12.1)	−0.044
*≤ 60*	24,066 (13.7)	23,046 (13.2)	
61–70	33,736 (19.3)	33,744 (19.3)	
71–80	61,573 (35.2)	58.043 (33.2)	
> 80	55,617 (31.8)	60.059 (34.4)	
Sex			
Female	84.765 (48.4)	85,194(48.7)	−0.002
Male	90,227 (51.6)	89,798 (51.3)	
Observation time prior to the index date (years), mean (SD)	9.0 (5.8)	8.9 (5.9)	0.016
Diagnoses documented prior to index date			
Obesity	24,737 (14.1)	23,539 (13.5)	−0.007
Diabetes mellitus	49.691 (28.4)	48,768 (27.9)	−0.005
Hypertension	120.378 (68.8)	122,481 (70.0)	0.012
Dyslipidemia	71.580 (40.9)	70.804 (40.5)	−0.004
Nicotine dependence	8,328 (4.8)	8,150 (4.7)	−0.002
COPD	23,486 (13.4)	21,521 (12.3)	−0.012
Ischemic heart diseases	49,767 (28.4)	46,451 (26.5)	−0.019
Heart valve diseases	17,393 (9.9)	14,530 (8.3)	−0.017
Heart failure	28.207 (16.1)	23,834 (13.6)	−0.026
Sleep disorders	28,793 (16.5)	26,826 (15.3)	−0.011
Treatments documented prior to index date			
Diuretics	59.080 (33.8)	54,249 (31.0)	−0.028
Betablockers	82.543 (47.2)	79,664 (45.5)	−0.016
Calcium-channel blockers	48,339 (27.6)	46,195 (26.4)	−0.012
ACE inhibitors	61,251 (35.0)	59,844 (34.2)	−0.008
Angiotensin II receptor blockers	47,037 (26.9)	44,910 (25.7)	−0.012
Lipid lowering drugs	57,898 (33.1)	56,002 (32.0)	−0.018
Thyroid hormones	31,133 (17.8)	29.841 (17.1)	−0.007
Systemic corticosteroids	21,366 (12.2)	19,421 (11.1)	−0.011
Beta-agonists	23,920 (13.7)	21,734 (12.4)	−0.012

Data are absolute samples and percentages unless otherwise specified

*Abbreviations* *AF *Atrial Fibrillation, *SMD *Standardized Mean Difference, *SD S*tandard Deviation, *COPD *Chronic Obstructive Pulmonary Disease, *ACE* Angiotensin Converting Enzyme

### Overall association between aids and AF

A diagnosis of an autoimmune disease (AID) before the index date was present in 11.3% of AF patients and 10.3% of controls. This corresponded to a statistically significant association between AID and AF (OR 1.12; 95% CI 1.10–1.15; *p* < 0.001).

### Associations with individual aids

**Fig. 3:**
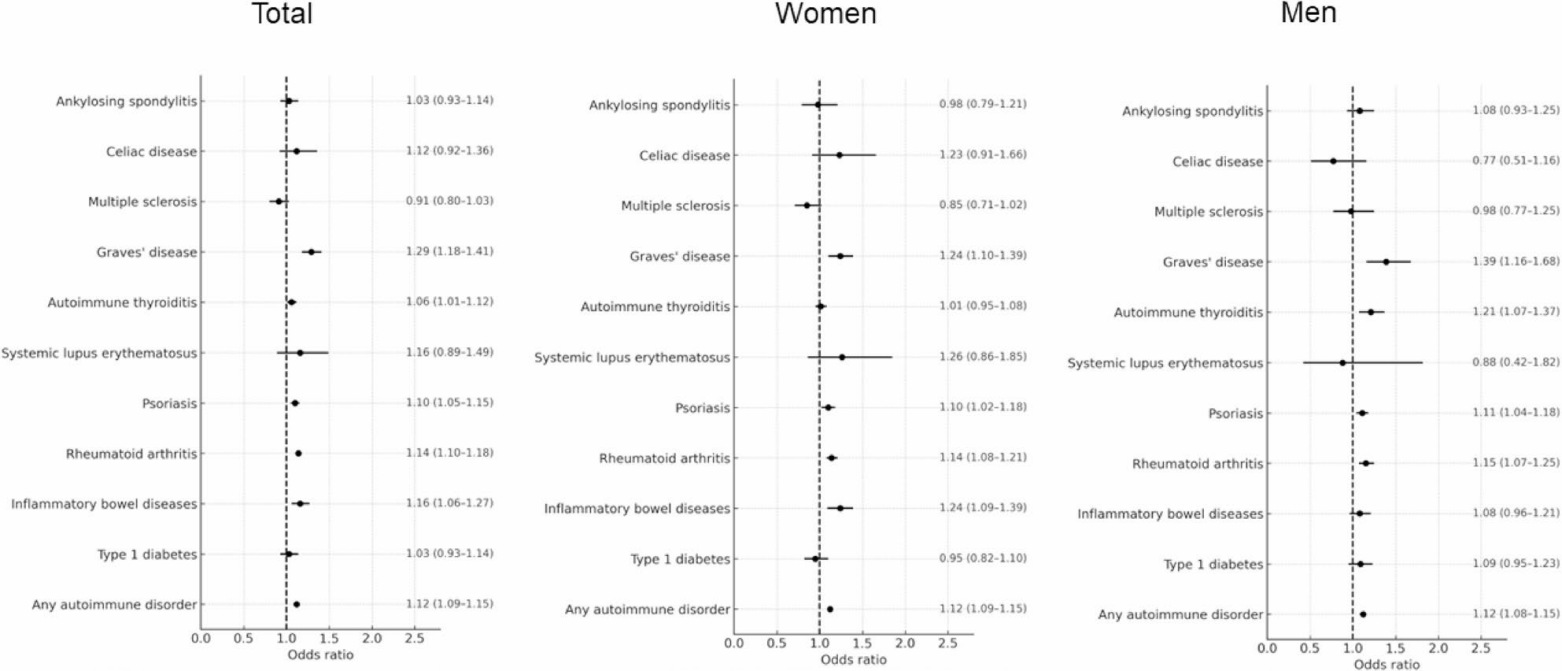
Association of various autoimmune diseases with atrial fibrillation in the total population and stratified by sex. (Forest Plot)

Several individual autoimmune conditions showed statistically significant associations with AF (Fig. 3). Rheumatoid arthritis was associated with higher odds of AF (OR 1.15; 95% CI 1.10–1.19; *p* < 0.001), as were inflammatory bowel diseases (OR 1.14; 95% CI 1.07–1.22; *p* < 0.001), psoriasis (OR 1.11; 95% CI 1.07–1.15; *p* < 0.001), and Graves’ disease (OR 1.29; 95% CI 1.18–1.41; *p* < 0.001).

Other AIDs—including type 1 diabetes, systemic lupus erythematosus, multiple sclerosis, celiac disease, autoimmune thyroiditis, and ankylosing spondylitis—did not show statistically significant associations with AF in the overall sample (Fig. 3).

### Sex-stratified analyses

**Fig. 4:**
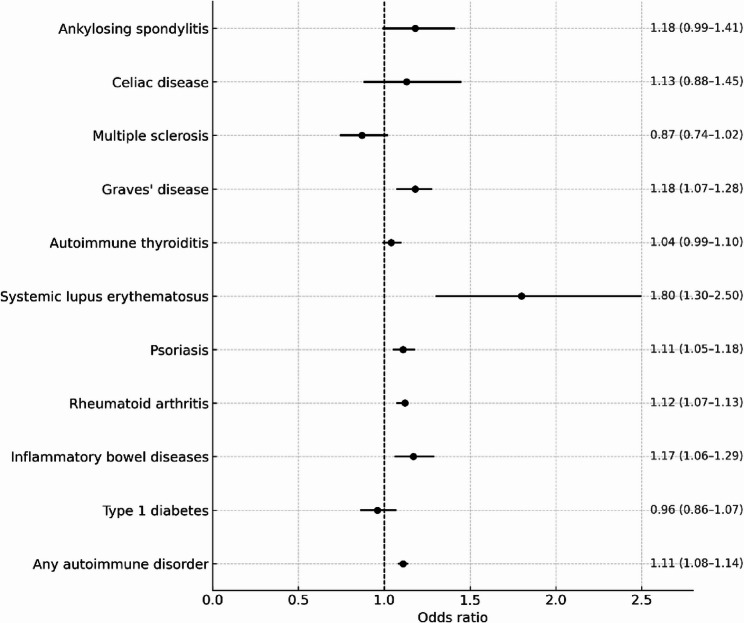
Association between autoimmune disorders and atrial fibrillation diagnosis – interaction of female sex and AID (Forest Plot).Interaction analysis assessing effect modification by sex on the association between autoimmune diseases and atrial fibrillation. Odds ratios represent sex × autoimmune disease interaction terms derived from conditional logistic regression models

Table 2 summarizes the proportion of patients with AID among AF cases and matched controls, overall and stratified by sex. Results from conditional logistic regression analyses, including sex-stratified odds ratios and sex × autoimmune disease interaction estimates, are presented in Figs. 3 and 4.

**Table 2:** Proportion of patients with autoimmune diseases among atrial fibrillation cases and matched controls, overall and stratified by sex

Variable	Total	Women			Men	
	Proportion of patients among AF cases (%)	Proportion of patients among controls (without AF) (%)	Proportion of patients among AF cases (%)	Proportion of patients among controls (without AF) (%)	Proportion of patients among AF cases (%)	Proportion of patients among controls (without AF) (%)
Any autoimmune disorder	11.34	10.26	13.59	12.31	9.22	8.34
Type 1 diabetes	0.82	0.78	0.73	0.70	0.91	0.86
Inflammatory bowel diseases	1.06	0.92	1.11	0.91	1.01	0.92
Rheumatoid arthritis	3.52	3.06	4.66	4.08	2.44	2.10
Psoriasis	3.33	2.99	3.11	2.79	3.53	3.18
Systemic lupus erythematosus	0.07	0.05	0.12	0.09	0.02	0.02
Autoimmune thyroiditis	2.11	2.03	3.46	3.38	0.85	0.75
Graves’ disease	0.77	0.62	1.15	0.92	0.42	0.33
Multiple sclerosis	0.28	0.30	0.35	0.38	0.21	0.22
Celiac disease	0.11	0.10	0.15	0.14	0.07	0.08
Ankylosing spondylitis	0.46	0.42	0.33	0.30	0.59	0.54

*Abbreviations* *AF* Atrial Fibrillation

Sex-stratified analyses showed differences in point estimates for certain autoimmune diseases. However, these findings were interpreted in the context of the corresponding interaction analyses (Figs. 3 and 4). IBD was associated with AF among women (OR 1.24; 95% CI 1.09–1.39), but not among men (OR 1.08; 95% CI 0.96–1.21). Autoimmune thyroiditis was associated with AF in men (OR 1.21; 95% CI 1.07–1.37), but not in women (OR 1.01; 95% CI 0.95–1.08). Graves’ disease showed a significant association in both sexes, with a stronger association in men (OR 1.39; 95% CI 1.16–1.68) than in women (OR 1.24; 95% CI 1.10–1.39). Rheumatoid arthritis and psoriasis were consistently associated with AF in both sexes, and effect sizes were comparable. Full sex-specific results are shown in Figs. 3 and 4.

### Interaction analyses

Interaction analyses assessed whether the associations between AIDs and AF differed between women and men. For most AIDs, interaction ORs were close to 1.00, and the corresponding 95% CIs included unity, indicating no statistically significant modification by sex.

Two conditions showed signals of possible sex-related variation. SLE showed a higher interaction estimate (OR 1.80; 95% CI 1.30–2.50). However, given the very low prevalence of SLE in this dataset, this estimate may be statistically unstable and should be interpreted with caution. Ankylosing spondylitis demonstrated a borderline interaction (OR 1.18; 95% CI 0.99–1.41).

For all other AIDs—including type 1 diabetes, rheumatoid arthritis, psoriasis, autoimmune thyroiditis, Graves’ disease, multiple sclerosis, and celiac disease—no evidence of sex-specific differences in association was observed. Complete interaction estimates are displayed in Fig. 4.

## Discussion

In this large, retrospective matched cohort, we found that several AIDs were modestly but significantly associated with AF. Notably, rheumatoid arthritis, IBD, psoriasis, and Graves’ disease showed the strongest associations, with consistent findings across sexes for some conditions and differences in sex-stratified point estimates for others.

Our findings are in line with prior large-scale observational studies. In a nationwide Danish cohort, Sun et al. reported increased long-term incidence of AF across 28 AIDs, with an adjusted hazard ratio of 1.63 for AID patients compared to matched controls [7]. Similarly, an analysis of UK biobank data demonstrated increased AF risk in patients with several autoimmune conditions, particularly among women [8]. The present study contributes to the pool of evidence by using real-world outpatient data, which is relevant because both AIDs and AF are often managed on an outpatient basis. The results are of value as they were obtained by applying rigorous matching and sex-stratified analyses in a representative clinical population.

The observed associations may be explained by chronic systemic inflammation, a hallmark of many AIDs, which contributes to atrial fibrosis, electrical remodeling, and arrhythmogenesis [10, 11]. Elevated inflammatory biomarkers such as IL-6 have been shown to predict AF onset [12] and recent mechanistic studies suggest that T-cell–mediated immune responses may directly affect atrial tissue structure [13]. In this context, the consistent associations observed between AF and rheumatoid arthritis or psoriasis (both associated with persistent inflammatory activity) are biologically plausible.

In particular for Graves’ disease, the observed association with AF may partly reflect thyroid functional status, treatment patterns, and increased ascertainment intensity rather than autoimmunity per se. Given the use of outpatient ICD-10 coding, detailed information on disease activity, biochemical thyroid status, disease duration, and treatment response is not available, and residual confounding related to hyperthyroidism and its management cannot be excluded.

Furthermore, our finding of a significant association between IBD and AF, particularly in women, is supported by previous meta-analytic evidence [14]. The authors hypothesized that chronic systemic inflammation, endothelial dysfunction, and autonomic imbalance may contribute to this association.

The observed sex-specific patterns, such as the stronger association between IBD and AF in women and between autoimmune thyroiditis and AF in men, align with previous research highlighting sex differences in immune regulation and cardiovascular vulnerability [15, 16].

The observed sex-specific patterns should be interpreted with caution, as they reflect associations rather than confirmed biological differences. For most autoimmune diseases, interaction analyses did not support statistically significant effect modification by sex. Apparent differences in sex-stratified point estimates should therefore not be interpreted as true sex-specific effects in the absence of supporting interaction evidence. In addition, estimates for rare conditions such as systemic lupus erythematosus may be statistically unstable due to low prevalence and should be interpreted particularly cautiously.

Future research incorporating biological markers, such as sex hormone levels, hormone receptor activity, and detailed immunophenotyping, will be needed to explore potential sex-related pathways more directly.

In this matched outpatient cohort, patients with AIDs showed modestly increased odds of having AF, corresponding to an absolute prevalence difference of about 1% point. Although this represents a small relative association, even modest differences may be clinically relevant in older, multimorbid populations. Importantly, our findings reflect statistical associations rather than risk or incidence, and the effect size should be interpreted cautiously given the potential for residual confounding, misclassification, and surveillance bias.

Nevertheless, prior studies indicate that once AF is present, individuals with autoimmune diseases have a higher burden of AF-related complications, including thromboembolism, hemorrhage, heart failure and mortality, likely influenced by systemic inflammation and chronic target-organ involvement [17]. This broader clinical context suggests that patients with AIDs may benefit from earlier or more frequent rhythm surveillance, careful cardiovascular risk-factor optimization, and appropriately individualized anticoagulation strategies. Integrating awareness of AID status into AF detection pathways may therefore help identify unrecognized AF earlier and reduce the downstream impact of AF-related complications.

### Limitations

Several limitations should be acknowledged when interpreting our findings. First, due to the observational design and the use of routinely collected healthcare data, causal inference cannot be established, and residual confounding cannot be completely excluded. Diagnoses and prescriptions are documented only at the time of presentation or dispensing, which may not fully capture disease severity, duration, treatment adherence, or changes in clinical status over time. Furthermore, biomarkers and other clinical parameters were not, or only rarely, available and therefore could not be incorporated into the analyses. Similarly, sex-specific variables such as hormone therapy, menopausal status, pregnancy history, and parity were not available in this database and could not be included in the analyses. These missing variables introduce residual confounding. Lifestyle factors such as smoking, alcohol use, and low physical activity could bias associations away from the null, whereas the lack of information on disease severity or activity may bias estimates toward the null. As a result, the direction of bias may differ by condition, and the associations should be interpreted with caution.

Second, our data did not include information on ECG-confirmed AF diagnoses or cardiology referral codes. Identification of AF was based on diagnostic coding in routine clinical care, which is standard practice in epidemiological studies using insurance claims or administrative databases but may introduce some degree of misclassification. Likewise, measures of healthcare utilization—such as outpatient visit frequency prior to the index date—were not available. Although such variables could help evaluate potential surveillance or detection bias, the close matching of cases and controls, along with the balanced distribution of comorbidities and medication use, reduces the likelihood that differential healthcare contact substantially biased the observed associations.

Third, while additional sensitivity analyses—such as adjustment for healthcare utilization, restriction to ECG-confirmed AF, or the use of negative control outcomes—may provide further insights, these analyses could not be performed with the available data and would extend the scope of the present work beyond its primary aim. Our objective was to provide a focused and methodologically robust assessment of the association between AID and AF in a large, well-matched population rather than an exhaustive evaluation of all potential sources of surveillance bias. We have addressed key confounding structures through stringent matching and examined sex-specific associations through stratified and interaction analyses.

Finally, autoimmune diseases often manifest earlier in life, which may affect healthcare-seeking behaviour and diagnostic practices. This could influence the observed associations, particularly given the older age distribution of our study population. These factors may limit the generalizability of our findings to younger individuals or to rarer autoimmune conditions. Despite these limitations, the large sample size, comprehensive matching strategy, and consistent pattern of associations across multiple analytical approaches support the robustness of our findings.

### Clinical implications

Despite these limitations, our findings highlight that certain autoimmune diseases are modestly associated with AF in routine outpatient care. Patients with autoimmune conditions, particularly those characterized by chronic systemic inflammation, may therefore represent a clinically vulnerable subgroup in whom atrial fibrillation is more frequently observed.

In this context, heightened clinical awareness may facilitate timely recognition of AF once symptoms occur or clinical suspicion arises, enabling appropriate management in accordance with established guidelines to reduce AF-related complications such as stroke and heart failure.

Importantly, real-world data from Germany indicate that optimal management of atrial fibrillation remains challenging in elderly populations comparable to the cohort studied here. Data from a large emergency department–based atrial fibrillation registry have shown that inappropriate dosing of direct oral anticoagulants is common and associated with adverse clinical outcomes, particularly in older patients [18]. Similarly, analyses focusing on patients aged ≥ 80 years have demonstrated substantial gaps in anticoagulation quality and outcomes in routine clinical care [19].

Against this background, awareness of AID status may help clinicians identify particularly vulnerable subgroups among patients with AF who may benefit from closer clinical follow-up, careful cardiovascular risk-factor optimization, and appropriately individualized anticoagulation strategies in line with current guideline recommendations.

## Conclusion

AIDs are modestly but consistently associated with AF in routine outpatient care. These findings highlight the importance of chronic inflammation in the pathophysiology of AF and underscore the need for further studies to define the underlying mechanisms and to explore preventive strategies in high-risk subgroups.

## Data Availability

The data that support the findings of this study are derived from the Disease Analyzer database (IQVIA), which includes anonymized health records from general and specialist practices in Germany. The datasets used and analyzed during the current study are available from Prof. Kostev on reasonable request.

## Abbreviations

AF: Atrial fibrillation
AID: Autoimmune disease
CI: Confidence interval
COPD: Chronic obstructive pulmonary disease
DZHK: German Center for Cardiovascular Research
ICD-10: International Classification of Diseases
IBD: Inflammatory bowel disease
IQVIA: Institute for Human Data Science
OR: Odds ratio
SMD: Standardized mean difference
